# The Effect on Rat Thymocytes of the Simultaneous In Vivo Exposure to 50-Hz Electric and Magnetic Field and to Continuous Light

**DOI:** 10.1100/tsw.2004.183

**Published:** 2004-10-20

**Authors:** Daniela Quaglino, Miriam Capri, Luigi Zecca, Claudio Franceschi, Ivonne P. Ronchetti

**Affiliations:** ^1^ Department of Biomedical Sciences-General Pathology, University of Modena and Reggio Emilia, Via Campi 287, 4100 Modena, Italy; ^2^ Department of Experimental Pathology, University of Bologna, Via San Giacomo 14, 40126 Bologna, Italy; ^3^ Interdepartment Center L. Galvani, University of Bologna, Via S. Giacomo 12, 40126 Bologna, Italy; ^4^ Institute of Advanced Biomedical Technologies-CNR, Segrate, Milano, Italy

**Keywords:** thymus, rat, ELF-EMF, light, photic stress, morphology

## Abstract

Thymus plays an important role in the immune system and can be modulated by numerous environmental factors, including electromagnetic fields (EMF). The present study has been undertaken with the aim to investigate the role of long-term exposure to extremely low frequency electric and magnetic fields (ELF-EMF) on thymocytes of rats housed in a regular dark/light cycle or under continuous light. Male Sprague-Dawley rats, 2 months old, were exposed or sham exposed for 8 months to 50-Hz sinusoidal EMF at two levels of field strength (1 kV/m, 5 μT and 5 kV/m, 100 μT, respectively). Thymus from adult animals exhibits signs of gradual atrophy mainly due to collagen deposition and fat substitution. This physiological involution may be accelerated by continuous light exposure that induces a massive death of thymocytes. The concurrent exposure to continuous light and to ELF-EMF did not change significantly the rate of mitoses compared to sham-exposed rats, whereas the amount of cell death was significantly increased, also in comparison with animals exposed to EMF in a 12-h dark-light cycle. In conclusion, long-term exposure to ELF-EMF, in animals housed under continuous light, may reinforce the alterations due to a photic stress, suggesting that, *in vivo*, stress and ELF-EMF exposure can act in synergy determining a more rapid involution of the thymus and might be responsible for an increased susceptibility to the potentially hazardous effects of ELF-EMF.

### INTRODUCTION

A progressively expanded literature has been devoted in the last decades to the beneficial or noxious effects of electromagnetic fields (EMF) to live organisms[1,2,3,4]. Although several reports hypothesized a relationship between EMF exposure and cancer occurrence[3,5] and suggested that the immune system might be affected[6,7,8,9,10], both epidemiological investigations as well as experiments with laboratory animals frequently gave rise to contradictory results.

In previous *in vitro* experiments, it has been observed that exposure to extremely low frequency (ELF) EMF is not possibly affecting the functional characteristics of human lymphocytes[11,12]. However, the *in vivo* situation is certainly more complex and we have suggested that rat thymocytes may be regarded as targets of the *in vivo* prolonged exposure to ELF-EMF[6].

Furthermore, thymocytes can be severely affected by other environmental factors such as light[13]. Continuous light exposure, for instance, even for relatively short periods, may induce a depression of the immune responses causing thymus involution, reduction of both cortex and medulla, and loss of thymocytes[14].

We performed *in vivo* experiments in order to (1) add further information on the role of a prolonged exposure to continuous light on rat thymocytes and (2) evaluate the possibility that a photic stress may act in synergy with a long-term exposure to ELF-EMF.

### MATERIALS AND METHODS

#### Animals and Exposure System

Two-month-old, male Sprague-Dawley rats (Charles River, Calco, Italy) were housed in the CESI (Milan, Italy) animal care exposure facilities and were fed *ad libitum* in a dark-light cycle (12:12 h) or under continuous light. Animals were further divided into three groups that were sham exposed or exposed to 50-Hz sinusoidal EMF at two levels of field strength (1 kV/m, 5 μT and 5 kV/m, 100 μT), respectively. Exposure to EMF was performed for 8 months, 5 days/week (Monday through Friday), 8 h/day (9 a.m. to 5 p.m.). Rats were individually housed in polycarbonate cages placed on electrically grounded metal nets in three identical exposure units. The electric field was generated by parallel electrodes with a 40-cm air gap. The magnetic field was generated by five pairs of vertically arranged rectangular coils (width 1.9 m, height 0.95 m). The external size of the system was 1.9 × 1.9 m and the distance between two adjacent pairs of coils was 0.6 m. Magnetic coils were fixed to wooden frames and the absence of vibrations was confirmed by acceleration measurements. Frames with coils were mechanically separated from the one holding the animal cages. The coil geometry was optimized in order to reduce the joule-heating losses at a level not affecting the microenvironment temperature. Sham-exposed animals were held in a similar nonenergized system. In the sham unit, after energizing the system, the measured magnetic field corresponded to the background level (0.04 μT).

At least six animals from each experimental condition were sacrificed by decapitation between 9 a.m. and 12.30 p.m., then thymus was removed and processed for electron microscopy. No abnormalities were noted from the gross and anatomo-pathological points of view.

#### Morphology

At sacrifice, the left part of the thymus from each animal was cut into 1-$mm^3$ pieces, fixed in 2.5% glutaraldehyde in phosphate buffered saline (PBS) pH 7.4 for 24 h at room temperature, postfixed in 1% osmium tetroxide in the same buffer for 2 h at room temperature, dehydrated in alcohol, and embedded in epoxy resin. Sections, 1 μm thick, were stained with 1% toluidine blue and observed in a Zeiss Axiophot light microscope. Selected areas were further analyzed on ultrathin sections with a Jeol 1200 EX electron microscope.

### Morphometry

Semi-thin sections of at least five specimens containing lymphoid tissue from each animal were carefully observed by light microscopy. The resin embedded material shows an excellent resolution allowing the evaluation of details that cannot be appreciated by classical histology. Only specimens comprehensive of both cortex and medulla were considered. In order to homogeneously compare the trend of data obtained using different criteria of evaluation, i.e., frequency and appearance, scores from 0–5 have been attributed, at 40× magnification, by two different investigators to the following parameters: cell-cell adhesion, mitoses, and cell death. All data have been referred to 0.1 $mm^2$ of tissue. Mitoses and apoptoses were counted and scored on a semi-quantitative scale from 0–5 (0, absent; 1, from 1–5; 2, from 6–10; 3, from 11–15; 4, from 16–20; 5, more than 20 on random fields). Necrosis and clustered cell death were graded semi-quantitatively on a scale from 0–5 (0, absent; 1, very scarce but present; 2; scarce; 3; moderately represented; 4, relatively abundant; 5, exuberant). Cell-cell adhesion was evaluated according to the space between thymocytes and their shape; score 0 was given when thymocytes were round and spread in an abundant necrotic or fibrotic material; score 1 was attributed to round thymocytes only rarely in close apposition and separated by areas devoid of cells; score 2 was assigned when at least 70% of thymocytes were round and dissociated, while the others were polygonal and maintained contact among themselves; score 3 was ascribed when at least 50% of thymocytes were round and dissociated, while the others were polygonal and maintained contact among themselves; score 4 was given when the majority of thymocytes presented a polygonal shape with limited disconnection; score 5 was attributed when almost all thymocytes were polygonal, in close apposition one to the other.

Kruskal-Wallis and Dunn nonparametric statistical analyses were performed in order to assess the significance of our data.

## RESULTS

### Continuous Light Exposure

In 10-month-old animals, thymus exhibited the morphology typical of adult animals: the organ was surrounded by a collagen capsule penetrating into the parenchyma and exhibiting few fibroblasts and small blood vessels. Areas of lymphoid tissue were frequently replaced with lipid degeneration. The separation between cortex and medulla was not always well defined and cells appeared quite separated one from the others (Fig. 1a,c). Animals housed under continuous light (CL), compared with those caged in a dark-light cycle (DL), exhibited more pronounced degenerative features. For instance, thymocytes were heterogeneous in size and shape, exhibited a more frequently condensed chromatin; furthermore, macrophages, swallowed up by necrotic and apoptotic debris, were more numerous (Fig. 1b). At the ultrastructural level, thymocytes appeared more disconnected to each other and surrounded by abundant cell debris (Fig. 1d).

Morphometric analyses revealed that, compared to animals housed in a DL, those caged under CL did not exhibit significant changes in the amount of mitoses (Fig. 2a), showed a significant increase of apoptosis (Fig. 2b) as well as of necrosis (Fig. 2c), whereas clustered cell death[15] was remarkably reduced (Fig. 2d).

The more pronounced cell death (Fig. 2e), observed by light and electron microscopy in the thymus of photic stressed animals, was associated to a consistent reduction of cell-cell adhesion (Fig. 2f).

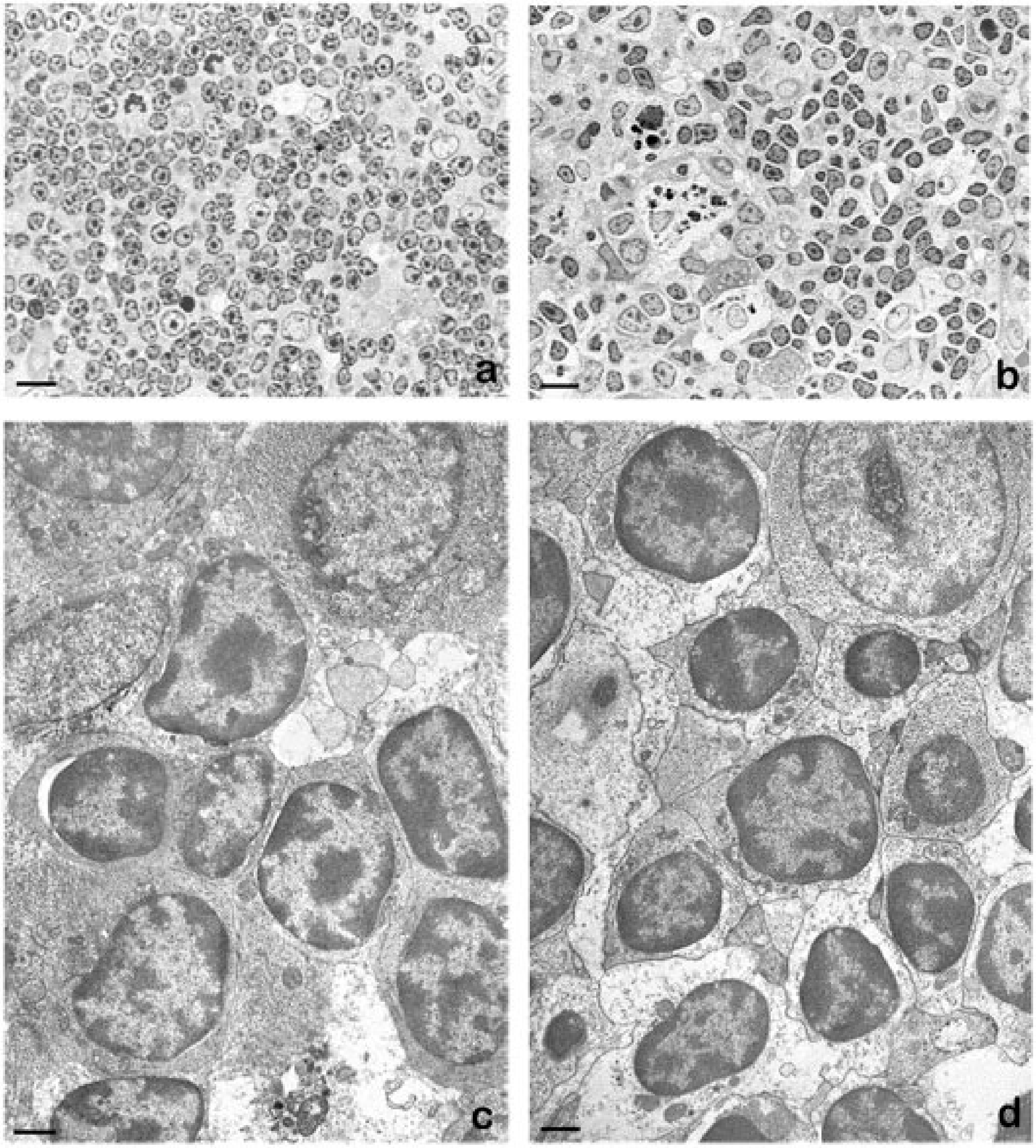

**FIGURE 1**. Light (a,b) and electron microscopy (c,d) of thymocytes from 10-month-old rats housed in a DL (a,c) or under CL (b,d). Exposure to CL increases the amount of degenerative features. Bar: 10 μm (a,b), 1 μm (c,d).

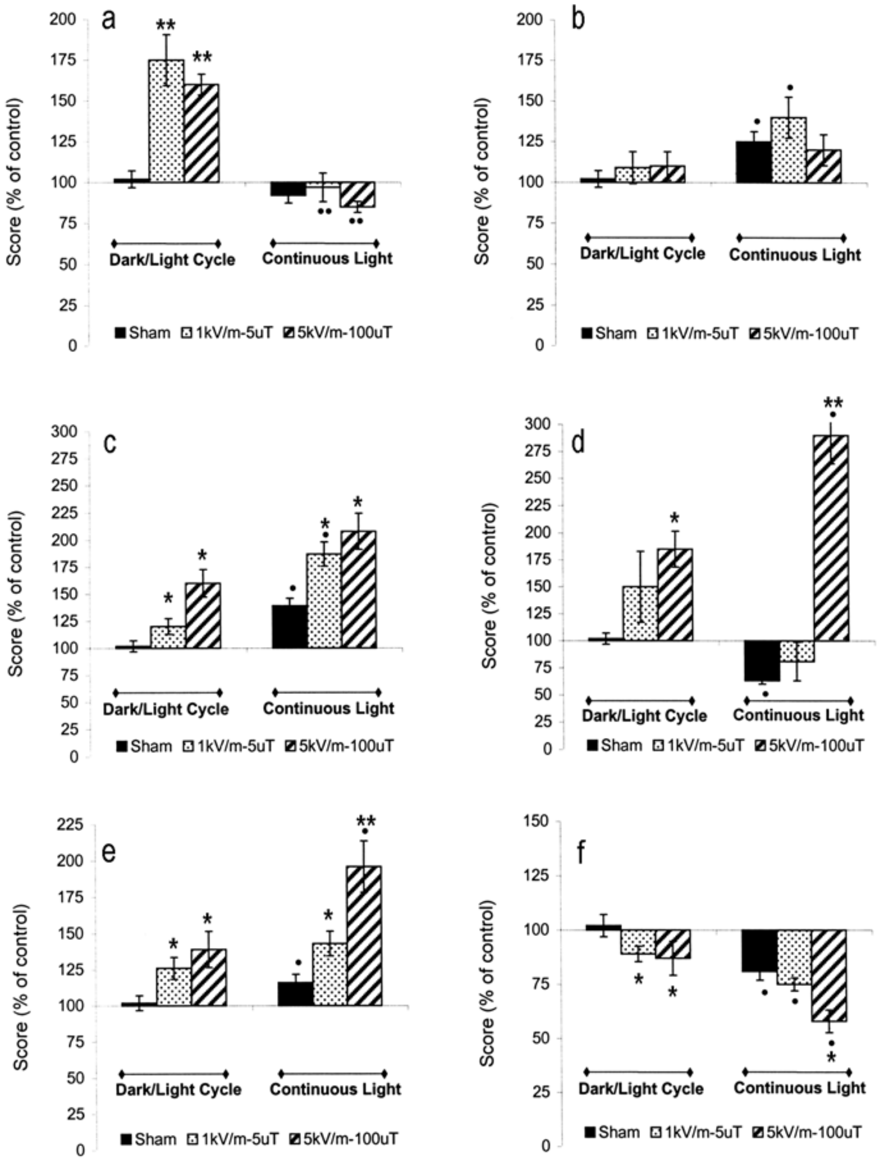

**FIGURE 2.** Morphometric analyses performed on semi-thin sections of thymus from rats housed in a DL or under CL. Rats were also sham exposed or exposed for 8 months to 50-Hz sinusoidal EMF at two levels of field intensity (1 kV/m, 5 μT and 5 kV/m, 100 μT). Mitoses (a), apoptosis (b), necrosis (c), clustered cell death (d), total cell death (e), and cell-cell adhesion (f) were scored semi-quantitatively (see also Materials and Methods) and data are expressed as percentage of variations compared to control sham-exposed rats housed in a DL ± SD. * $p < 0.05$; ** $p < 0.001$ significance vs. sham-exposed rats with the same light conditions. ● $p < 0.05$, ●● $p < 0.001$ significance vs. sham- or EMF-exposed rats in a DL.

### Continuous Light and ELF-EMF Exposure

Compared with animals caged under CL (Fig. 3a), those also exposed to ELF-EMF (Fig. 3b,c) exhibited several morphologic alterations such as an increased amount of cell death, especially necrosis and clustered cell death. In particular, the thymus of animals exposed to CL and to ELF-EMF (CL-ELF-EMF) showed evident areas of necrosis and clustered cell death especially at the highest field intensity (Fig. 3c). These features were clearly observed also at the ultrastructural level where thymocytes, surrounded by abundant cell debris, exhibited signs of distress such as vacuolizations, chromatin condensation, and clustered cell death (Fig. 4).

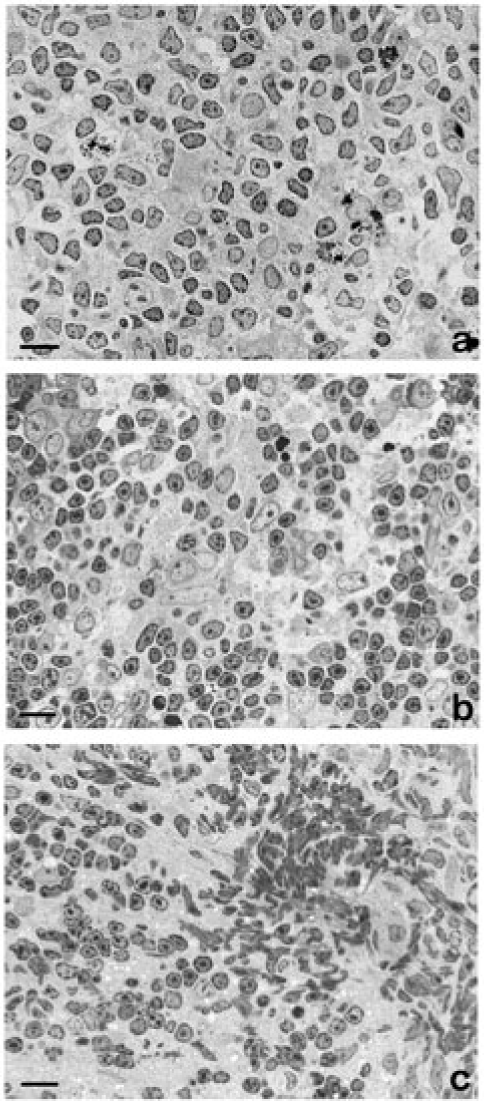

**FIGURE 3**. Light microscopy of thymus from 10-month-old rats exposed for 8 months to CL (a) and to 50-Hz sinusoidal EMF at two levels of field intensity (b, 1 kV/m, 5 μT and c, 5 kV/m, 100 μT). Cell death, in particular necrosis and clustered cell death, was more pronounced after EMF exposure (b,c). Bar: 10 μm.

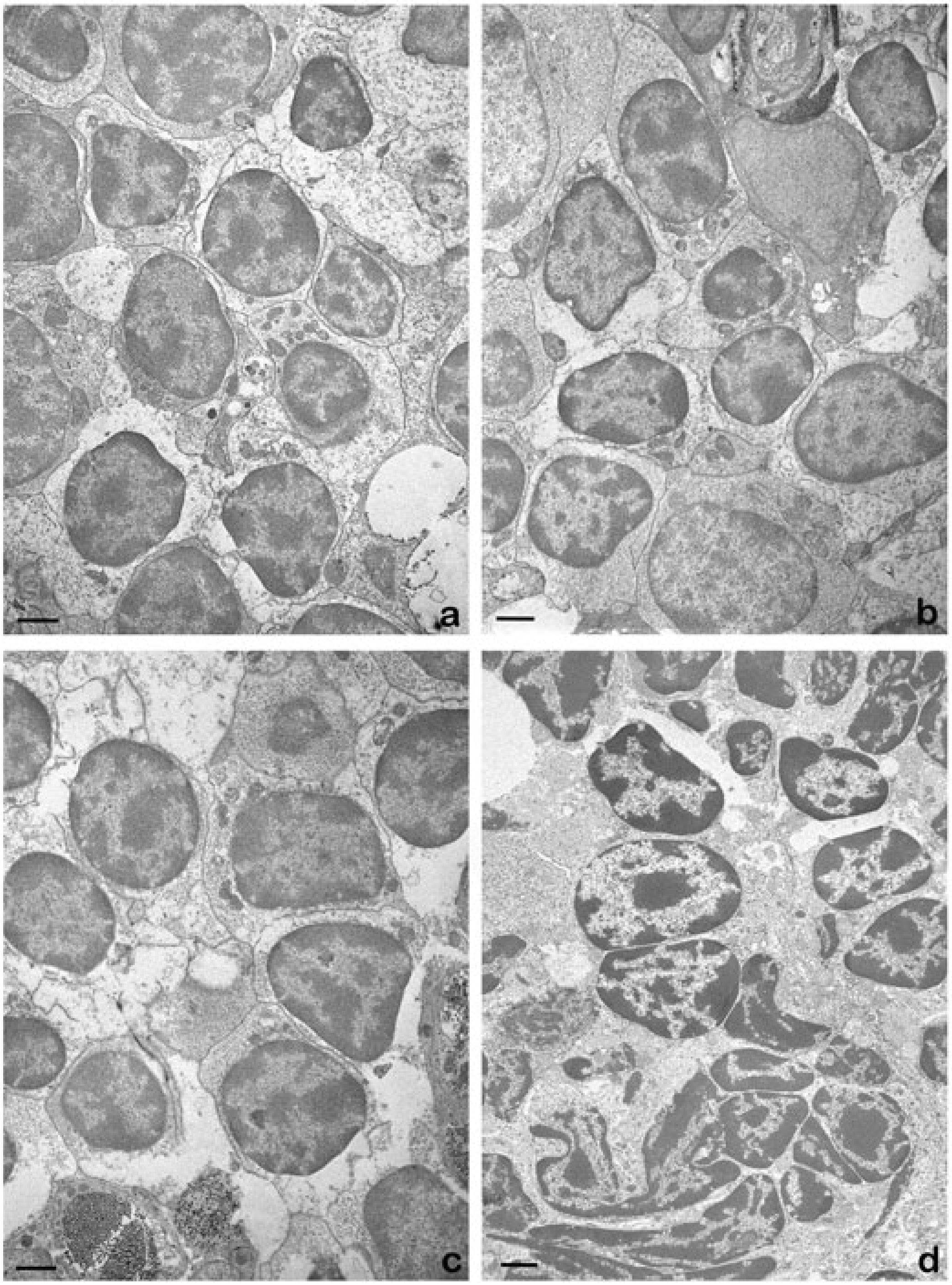

**FIGURE 4**. Electron microscopy of thymus from 10-month-old rats exposed for 8 months to CL (a) and to 50-Hz sinusoidal EMF at two levels of field intensity (b, 1 kV/m, 5 μT and c,d, 5 kV/m, 100 μT). Necrosis and clustered cell death are clearly responsible for the dramatic reduction of cell-cell adhesion in animals exposed under CL to EMF. Bar: 1 μm.

Morphometric analyses, comparing rats exposed to CL-ELF-EMF with those exposed to DL-ELF-EMF and with sham-exposed animals, revealed that mitoses were significantly increased only after DL-

ELF-EMF exposure (Fig. 2a), whereas apoptoses appeared to increase only in CL-ELF-EMF exposure, at the lower field strength (Fig. 2b).

Interestingly, necrosis was dramatically increased in all CL-ELF-EMF–exposed animals proportionally to field intensity (Fig. 2c); moreover, this increment was more evident in these rats compared to those exposed in DL.

CL-ELF-EMF exposure determined, at the higher field intensity, a conspicuous increase of clustered cell death, thus reverting the decline exerted by the photic stress alone (Fig. 2d).

Furthermore, cell-cell adhesion, that was already decreased in DL-ELF-EMF–exposed rats, was even more dramatically reduced in CL-ELF-EMF–exposed animals (Fig. 2f). These changes appeared to be dependent on field strength and to the increase of total cell death (Fig. 2e).

### CONCLUSIONS

Continuous light has been regarded as an environmental stress that can play a role in the morphofunctional modification of the neuroimmunological endocrine system, at least in the rat model[16,17]. It has been recently demonstrated that a 1-month period of permanent light causes structural changes to the rat thymus such as weight decrement, reduced cellularity, and increased apoptosis, especially in the cortex. These data suggested that stress can be responsible for a thymic involution, although reversible, thus impairing some immunological functions[14]. We have already suggested that thymus, even in adult animals, may be a target of an *in vivo* prolonged exposure to ELF-EMF, thus modulating cellular turnover and favoring thymic involution[6].

The present study has been performed on the rat model in order to add further information regarding the role of a prolonged exposure to permanent light on thymus morphology and to evaluate the potential synergic effect of a stress condition and of ELF-EMF exposure.

Results confirm that photic stress is able to induce several alterations by increasing, for instance, the level of cell death. Mahmoud et al.[14] focused on the increased number of apoptoses in the thymus of animals exposed under continuous light. In the present study, despite the different period of exposure (1 vs. 8 months) and/or the age of the animals (3 vs. 10 months), we obtained similar results, indicating that apoptosis may be regarded as a general response of thymocytes to stress conditions.

Moreover, continuous light exposure may also dramatically increase necrosis, whereas clustered cell death was diminished. Results obtained in these experiments, together with the decreased cell-cell adhesion, are consistent with an accelerated thymic involution in those animals that have been caged under continuous light.

When animals were also exposed to ELF-EMF, the increased number of mitoses, normally observed after ELF-EMF exposure alone[6], was never noticed. By contrast, the combination of photic and electromagnetic stress determined an increased cell death. While the lowest field intensity seems to induce apoptoses more preferentially, the highest field intensity interferes with thymocytes mainly through necrosis and clustered cell death, suggesting once more that thymocytes may die through at least three different ways[13,15,18].

In conclusion, continuous light and ELF-EMF exposure can interfere with thymus morphology by themselves as well as in combination. The synergic effect of the combination of the two stresses has been demonstrated by comparing data from the CL-ELF-EMF–exposed animals vs. sham-exposed rats housed in a 12-h dark-light cycle or under continuous light. Moreover, these effects were, generally, related to field strength.

Although further investigations, verifying the real efficiency of the immune system, are required before extrapolating these results to human beings, these data indicate that, *in vivo*, a photic stress and a ELF-EMF exposure determine a more rapid involution of the thymus and might be responsible for an increased susceptibility to the suspected carcinogenic-promoting effects of ELF-EMF[19]. This has been recently suggested in a study where different light regimens, in association with EMF exposure, have been demonstrated to interfere with mammary carcinogenesis in the rat model[20].

### ACKNOWLEDGMENTS

Work supported by grants from CNR-ENEL: Interaction of energetic systems with human health and environment, MURST ex-60%, and by the Fondazione del Monte di Bologna e Ravenna (2002-2004).
